# Linagliptin Attenuates the Cardiac Dysfunction Associated With Experimental Sepsis in Mice With Pre-existing Type 2 Diabetes by Inhibiting NF-κB

**DOI:** 10.3389/fimmu.2018.02996

**Published:** 2018-12-18

**Authors:** Sura Al Zoubi, Jianmin Chen, Catherine Murphy, Lukas Martin, Fausto Chiazza, Debora Collotta, Muhammad M. Yaqoob, Massimo Collino, Christoph Thiemermann

**Affiliations:** ^1^Centre for Translational Medicine and Therapeutics, William Harvey Research Institute, Queen Mary University of London, London, United Kingdom; ^2^Department of Drug Science and Technology, University of Turin, Turin, Italy

**Keywords:** sepsis, septic cardiomyopathy, NF-κB, IKK-16, DPP-4, linagliptin, type 2 diabetes mellitus, mice

## Abstract

The mortality rate of patients who develop sepsis-related cardiac dysfunction is high. Many disease conditions (e.g., diabetes) increase the susceptibility to infections and subsequently sepsis. Activation of the NF-κB pathway plays a crucial role in the pathophysiology of sepsis-associated cardiac dysfunction and diabetic cardiomyopathy. The effect of diabetes on outcomes in patients with sepsis is still highly controversial. We here hypothesized that type 2 diabetes (T2DM) augments the cardiac (organ) dysfunction associated with sepsis, and that inhibition of the NF-κB pathway with linagliptin attenuates the cardiac (organ) dysfunction in mice with T2DM/sepsis. To investigate this, 10-week old male C57BL/6 mice were randomized to receive normal chow or high fat diet (HFD), 60% of calories derived from fat). After 12 weeks, mice were subjected to sham surgery or cecal ligation and puncture (CLP) for 24 h. At 1 hour after surgery, mice were treated with linagliptin (10 mg/kg, i.v.), IKK-16 (1 mg/kg, i.v.), or vehicle (2% DMSO, 3 ml/kg, i.v.). Mice also received analgesia, fluids and antibiotics at 6 and 18 h after surgery. Mice that received HFD showed a significant increase in body weight, impairment in glucose tolerance, reduction in ejection fraction (%EF), and increase in alanine aminotransferase (ALT). Mice on HFD subjected to CLP showed further reduction in %EF, increase in ALT, developed acute kidney dysfunction and lung injury. They also showed significant increase in NF-κB pathway, iNOS expression, and serum inflammatory cytokines compared to sham surgery group. Treatment of HFD-CLP mice with linagliptin or IKK-16 resulted in significant reductions in (i) cardiac, liver, kidney, and lung injury associated with CLP-sepsis, (ii) NF-κB activation and iNOS expression in the heart, and (iii) serum inflammatory cytokine levels compared to HFD-CLP mice treated with vehicle. Our data show that pre-existing type 2 diabetes phenotype worsens the organ dysfunction/injury associated with CLP-sepsis in mice. Most notably, inhibition of NF-κB reduces the organ dysfunction/injury associated with sepsis in mice with pre-existing T2DM.

## Introduction

Sepsis is a dysregulated body response to infection that results in life-threatening organ dysfunction (1). The cardiovascular system is one of the important systems affected by sepsis. Most septic patients, and all patients with septic shock, develop sepsis-related cardiovascular dysfunction (2, 3), which is a key driver of in-hospital mortality in these patients (4). Both the incidence of sepsis and sepsis-related mortality increase with age due to the presence of significant comorbidities including chronic kidney disease and type 2 diabetes in the elderly (5). The prevalence of diabetes is increasing worldwide (6). Patients with diabetes are at high risk of developing diabetic cardiomyopathy, left ventricular (LV) hypertrophy, ischemic cardiac injury, and heart failure (6–9). Diabetic patients are more susceptible to both common and rare infections and have a higher incidence of sepsis than patients that do not suffer from the disease (10, 11). However, the effect of diabetes on outcome in patients with infections is controversial with some studies showing increased hospitalization, organ dysfunction/injury, and mortality in diabetic patients with e.g., pneumonia (12–14), while other studies report either no effect (15–17) or even protective effects (18–21). Hence, this study was undertaken to investigate the effects of pre-existing type 2 diabetes mellitus (T2DM) caused by high fat diet (HFD) on cardiac dysfunction in mice with sepsis.

Dipeptidyl peptidase-4 (DPP-4) inhibitors (gliptins) are among the most recently approved drugs for the treatment of hyperglycemia in patients with T2DM. Gliptins mediate their anti-diabetic effects by primarily inhibiting degradation of endogenous glucagon-like peptide 1 (GLP-1) and glucose-dependent insulinotropic peptide (GIP), resulting in prolongation of postprandial insulin secretion. Given the numerous and varied substrates enzymatically cleaved or bound by DPP-4, DPP-4 inhibitors may have the potential to exhibit a broader range of salutary pleiotropic effects in the heart and vasculature, increasing the concentration of peptides with potential vasoactive and cardioprotective effects, which may be independent of GLP-1 and its receptor. DPP-4 signaling cascade has been recently demonstrated to be involved also in the pathologic features of sepsis, mainly due to a selective cross-talk between DPP-4 and nuclear factor- kappa B (NF-κB) signaling pathways (22–25). In sepsis, microbial components activate different antigen presenting cells (APCs) after binding to their Toll like receptor (TLR) 4 and 2. Exposed caveolin-1 from the activated APC interacts with DDP-4 in T cells which results in strong NF-κB activation in both the APCs and the T cells (23). Adenosine deaminase (ADA) is another activator of DPP-4. The activity of ADA in the serum is increased during inflammatory diseases (e.g., sepsis) as a result of increased macrophages activity (26) and the interaction of ADA with DPP-4 leads to NF-κB activation in T cells (22). The expression of DPP-4 is increased in diabetic patients (27–29).

Activation of NF-κB plays a crucial role in the pathophysiology of both septic (30–32) and diabetic cardiomyopathy (33). In sepsis, activation of NF-κB is secondary to activation of TLR 2 and 4 by e.g., by wall fragments of Gram-negative (e.g., lipopolysaccharide; LPS) or Gram-positive bacteria (e.g., peptidoglycan; PepG) and/or pro-inflammatory cytokines including tumor-necrosis factor-α (TNF-α) or interleukin-1 (IL-1). In addition to pro-inflammatory cytokines, the activation of NF-κB in diabetes is also driven by free fatty acids (34) (which activate TLR4) and advanced glycation end products (which activate RAGE) (35). We have recently demonstrated that inhibition of the activation of NF-κB by a selective IκB kinase (IKK) inhibitor (IKK-16) attenuates the cardiac dysfunction caused by sepsis in mice without co-morbidities (32) and in mice with pre-existing chronic kidney disease (36). However, the potential protective effects of the impairment of the cross-talk between DDP-4 and NF-κB activation in sepsis-induced multiorgan dysfunction have never been tested in animal models of diet-induced diabetes, which is known to be characterized by an increase in baseline NF-κB activity. Here we investigate (a) the role of NF-κB activation in the cardiac dysfunction caused by HFD with or without sepsis, (b) the effect of linagliptin treatment on cardiac performance in the model of sepsis and T2DM. To investigate the relative contribution of NF-κB inhibition in the observed effects of linagliptin, IKK-16, a potent and selective IKK inhibitor, was used as a comparative pharmacological tool.

## Materials and Methods

### Ethical Statement

The animal protocols followed in this study were approved by the local Animal Use and Care Committee in accordance with the derivatives of both, the Home Office Guidance in the Operation of Animals (Scientific Procedure Act 1986) published by Her Majesty's Stationary Office, the Guide for the Care and Use of Laboratory Animals of the National Research Council (home office project license, PPL: 70/8350) and by the local ethical committee (DGSAF 0021573-P-12/11/2013) and are in keeping with the European Directive 2010/63/EU as well as the 2011 Guide for the Care and Use of Laboratory Animals.

### Animals

This study was conducted on 56 male C57BL/6 mice aged 10 weeks (Charles River, Kent, UK) weighing 25–30 g, receiving a standard diet and water *ad libitum* (before starting the experiments). Mice were housed 5 per cage in a temperature-controlled room with a 12-h light/dark cycle.

### High Fat Diet Model of Type 2 Diabetes Mellitus

In this model of diet-induced obesity and insulin resistance, mice (Charles River UK, Kent) were randomized to receive standard chow diet [LabDiet®, St. Louis (5053: protein provides 25%, fat 13%, and carbohydrate 62% of the total calories)] or high fat diet [TestDiet®, St. Louis (58Y1: Blue diet; protein provides 18.1%, fat 61.6%, and carbohydrate 20.3% of the total calories)] with free access to water for 12 weeks. Body weight, food intake, and calories intake were measured weekly through the experiment to monitor feeding behavior.

### Oral Glucose Tolerance Test (OGTT)

Mice were fasted for 6 h with free access to water. At the end of the 6 h fasting, the body weight and fasting blood glucose were measured using tail vein blood. Mice then received a bolus dose of glucose (2 g/kg, dissolved in drinking water) via oral gavage. Blood glucose levels were then measured at 15, 30, 45, 60, 90, and 120 min post glucose administration using blood glucose meter Accu-Chek® (Accu-Chek Compact System; Roche Diagnostics, Basel, Switzerland).

### Insulin Tolerance Test (ITT)

Mice were fasted for 4 h with free access to water. At the end of the 4 h fasting, the body weight and fasting blood glucose were measured using tail vein blood. Mice then received a dose of insulin aspart (NovoRapid®) (0.75 U/kg, i.p.). Blood glucose level was then measured at 15, 30, 45, 60, 90, and 120 min post insulin administration using blood glucose meter Accu-Chek® (Accu-Chek Compact System; Roche Diagnostics, Basel, Switzerland).

### Measuring Fasting Plasma Insulin

Mice were fasted for 6 h with free access to water. At the end of the 6 h fasting period, blood samples were collected from the tail vein. Fasting plasma insulin levels were then measured using human insulin ELISA kit following the manufacturer's instructions (Abcam®, Cambridge, UK).

### Assessment of Baseline Kidney Function

During the last week of the experiment, mice were housed in the metabolic cages to collect urine. They were housed (one mouse per cage) for 24 h with free access to food and water. Urine biochemistry (creatinine and sodium levels) was assessed blindly by IDEXX the commercial veterinary testing laboratory (IDEXX Ltd; West Sussex, UK). Urine albumin was measured using a mouse albumin ELISA kit following manufacturer instructions (Cambridge Bioscience®, Cambridge, UK). Then creatinine clearance (CrCl) and urine albumin to creatinine ratio (ACR) were calculated using the following equations:

$$\begin{array}{l} \text{CrCl}=\frac{\text{Urine Creatinine}}{\text{Serum Creatinine}}\times \frac{\text{Urine Volume}}{\text{Time}} \end{array}$$

**Equation 1:** Creatinine clearance (ml/min) is calculated using four measurements (a) urine creatinine (μmol/L), (b) serum creatinine (μmol/L), (c) urine volume (ml), and (d) time (minutes).

$$\begin{array}{l} \text{ACR}=\;\frac{\text{Urine Albumin}}{\text{Urine Creatinine}} \end{array}$$

**Equation 2:** Urine albumin to creatinine ratio is calculated using 2 (a) urine albumin (μg/L) and (b) urine creatinine (mg/L).

### Assessment of Cardiac Function *in vivo* (Echocardiography)

Echocardiography was conducted *in vivo* at baseline (before sepsis challenge) then at 24 h after CLP to measure cardiac function using a 30 MHz RMV707B scan head and a Vevo-770 imaging system (VisualSonics, Toronto, Ontario, Canada).

Animals were anesthetized using 3% isoflurane delivered with 0.4 l/min oxygen in the anesthesia chamber. After being sedated, mice were then transferred to the echo table and taped from the limbs in a supine position onto the metal ECG leads on the Echo platform. Anesthesia was maintained during the whole imaging process using 0.5–2% isoflurane delivered with 0.4 l/min oxygen via nosecone under spontaneous breathing. The handling platform was warmed to 40°C in order to keep the core body temperature of the mice. After being placed on the platform, the fur on the chest was then removed carefully using Veet® hair removing cream. A pre-warmed echo gel is then applied to the chest to start the measurement. At least 10 min were left for the animals to stabilize before any measurement was taken. The body temperature was monitored using a rectal probe and the heart rate was obtained from ECG tracing during the whole procedure.

Measurements from both two-dimensional (brightness mode, B-mode) and one-dimensional (motion mode, M-mode) were obtained. Measurements of the left ventricle internal dimension (LVID) in both systole and diastole from the M-mode at the level of the papillary muscles were used to calculate the percentage ejection fraction (% EF), fractional shortening (% FS), and the measurements of LV end-systolic and end-diastolic areas from the B-mode were used to calculate the percentage functional area change (% FAC) using the following equations:

$$\begin{array}{l} \;\%\text{ EF }=\;\frac{\left(\text{LVID};{\text{d}}^{3}-\text{LVID};{\text{s}}^{3}\right)}{\text{LVID};{\text{d}}^{3}}\times 100 \end{array}$$

**Equation 3:** Ejection fraction (%) is calculated using 2 measurements (a) left ventricle internal dimension during diastole (LVID;d, mm) and (b) left ventricle internal dimension during systole (LVID;s, mm).

$$\begin{array}{l} \;\%\text{ FS }=\;\frac{\left(\text{LVID};\text{d}-\text{LVID};\text{s}\right)}{\text{LVID};\text{d}}\;\times 100 \end{array}$$

**Equation 4:** Fractional shortening (%) is calculated using 2 measurements (a) left ventricle internal dimension during diastole (LVID;d, mm) and (b) left ventricle internal dimension during systole (LVID;s, mm).

$$\begin{array}{l} \;\%\text{ FAC }=\;\frac{\left(\text{LV area};\text{d}-\text{LV area};\text{s}\right)}{\text{LV area};\text{d}}\;\times 100 \end{array}$$

**Equation 5:** Fractional area change (%) is calculated using 2 measurements (a) left ventricle end-diastolic area (LV area;d, mm^2^) and (b) left ventricle end-systolic area (LV area;s, mm^2^).

### Model of Cecal Ligation and Puncture (CLP) Induced Polymicrobial Sepsis

At 12 weeks after starting the HFD, mice fed either chow or HFD were randomized to undergo either sham operation or CLP surgery. Before surgery, mice were anesthetized using (1.5 ml/kg, i.p.) of 2:1 ketamine (100 mg/ml): xylazine (20 mg/ml) solution. To obtain adequate analgesia, buprenorphine (0.05 mg/kg, i.p.) was administered just before starting the surgery. The fur of the abdomen was removed using Veet® hair removing cream and the area cleaned with 70% ethanol. A 1.5 cm midline incision of the abdomen was made and the caecum was exposed. The caecum then was ligated below the ileocecal valve and two punctures were made one at each end using an 18-G needle. A small amount of fecal matter was then squeezed from both punctures before the caecum was returned to its anatomical position and the cut in the abdomen was then sutured. Each mouse then received a resuscitation fluid (1 ml 0.9% NaCl, s.c.). Mice were left on a homeothermic blanket to recover then placed back into fresh clean cages. At 1 h after CLP surgery, mice from the HFD group were randomized to receive linagliptin (10 mg/kg, i.v), IKK-16 (1 mg/kg i.v.) or vehicle (2% DMSO; 3 ml/kg, i.v.). At 6 and 18 h after surgery, antibiotic (imipenem/cilastatin, 20 mg/kg) dissolved in resuscitation fluid (15 ml/kg 0.9% NaCl, s.c.) was administered along with analgesia (buprenorphine, 0.05 mg/kg, i.p.). At 24 h, mice were anesthetized for assessment of cardiac function *in vivo*. As a terminal procedure, mice were anesthetized using high dose isoflurane (3% delivered in 0.9 l/min O_2_) before being sacrificed. Blood samples were collected by cardiac puncture and vital organs were collected and snap frozen using liquid nitrogen then stored for further analysis at −80°C freezer. Mice that underwent sham surgery were anesthetized and handled in the same manner as CLP mice during surgery. However, in animals undergoing sham surgery, the cecum (although exposed) was not subjected to perforation. At 1 h after surgery, sham mice were treated with vehicle (2% DMSO, 3 ml/kg, i.v.) and they were also treated with antibiotic (imipenem/cilastatin, 20 mg/kg) dissolved in resuscitation fluid (15 ml/kg 0.9% NaCl, s.c.) along with analgesia (buprenorphine, 0.05 mg/kg, i.p.) at 6 and 18 h after surgery. Surgery, treatments and assessment of cardiac function were performed by different member of the research team to minimize subjective errors. The investigator assessing the cardiac function was blinded as to the intervention that was used in the study.

### Western Blot Analysis

Immunoblot analyses of heart biopsies were carried out using a semi-quantitative western blotting. We measured the degree of phosphorylation of IKK, IκBα, and Akt, nuclear translocation of p65 NF-κB subunit to the nucleus and inducible nitric oxide synthase (iNOS) expression. For sample handling, blood was withdrawn from mice at the time of euthanasia, heart tissue was then washed with saline solution prior to homogenisation. For sample preparation, a piece of heart tissue was taken and diluted (1:10) with homogenisation buffer (HB) at 4°C to obtain a whole tissue lysate, that contains endothelium, cardiomyocytes and leucocytes, for protein extraction. Cytosolic and nuclear extracts from hearts were prepared as previously described (37). Succinctly, hearts were homogenized at 10% (wt/vol) with a Potter Elvehjem homogenizer (Wheaton, Millville, NJ) using a homogenization buffer containing 20 mM HEPES (pH 7.9), 1 mM MgCl_2_, 0.5 mM EDTA, 1% Nonidet P-40, 1 mM EGTA, 1 mM DTT, 0.5 mM PMSF, 1 μl/ml of PIC. Homogenates were centrifuged at 1,300 g for 5 min at 4°C. Supernatants were removed and centrifuged at 16,000 g at 4°C for 40 min to obtain supernatant containing the cytosolic fraction. The pelleted nuclei were resuspended in extraction buffer (1/3 volume of the homogenation buffer) containing 20 mM HEPES (pH 7.9), 1.5 mM MgCl2, 300 mM NaCl, 0.2 mM EDTA, 20% glycerol, 1 mM EGTA, 1 mM DTT, 0.5 mM PMSF, 1 μl/ml of PIC, and incubated in ice for 30 min, followed by centrifugation at 16,000 g for 20 min at 4°C. The resulting supernatants containing nuclear proteins were carefully removed. Both cytosolic and nuclear proteins were measured using bicinchoninic acid (BCA) protein assay following manufacturer's directions (Therma Fisher Scientific, Rockford, IL). Proteins were separated by gel electrophoresis using sodium dodecyl sulfate polyacrylamide (SDS-PAGE) then transferred into a Polyvinylidene difluoride (PVDF) membrane. The membrane then was blocked 1 h in 5% dry milk solution in TBS-tween). Incubation of the membrane was conducted overnight at 4°C with primary antibody in 5% blocking solution then incubated the next day with the appropriated HRP-conjugated secondary antibody at room temperature for 30 min and then detected with enhanced chemilumescent (ECL) detection system and quantified by densitometry using BioRad Image Lab Software^TM^ 6.0. Results were normalized with respect to densitometric value of mouse anti-tubulin for cytosolic and total proteins, and mouse anti-histone H3 for nuclear proteins.

### Cytokine Measurements

Serum cytokines levels (TNF-α, IL-6, KC, and IL-10) were measured using a bead-based immunoassay method. Serum samples were prepared and handled following manufacturer instructions (Biolegend®, San Diego, USA). Data was obtained using a LSR Fortessa (Biociences®, Berkshire, UK) and analyzed using the Legendplex^TM^ 7.1.0.0 software.

### Measuring Myeloperoxidase (MPO) Activity in the Lung

MPO was extracted from the tissues according to the methods described by Barone et al. (38) with slight modifications to measure neutrophil accumulation in the lungs. For samples preparation, a piece of lung was diluted (1:20) with 5 mM potassium phosphate buffer to homogenize the sample (at 4°C). For measurements of MPO activity, the homogenate was centrifuged (at 13,000 RPM, 30 min, 4°C). The resulted supernatant was discarded. A 5-time dilution with 0.5% hexadecyl-trimethyl-ammonium bromide in 50 mM potassium phosphate buffer was used to suspend and homogenize the pellet. The resulted solution was then frozen and thawed three times followed by 10 s sonication at room temperature and then incubated at 4°C for 30 min then centrifuged (at 12,500 RPM, 15 min, 4°C). MPO activity was measured in the supernatant by mixing 100 μl of the supernatant with 0.167 mg/ml o-dianiside dihydrochloride and 0.0005% hydrogen peroxide in 2.9 ml 50 mM potassium phosphate buffer. UV-visible spectrophotometer was used to measure the change in absorbance at 460 nm for 1.5 min. MPO activity was presented as the quantity of the enzyme degraded 1 μmol of peroxide/min at 25°C and expressed as μU/gram of the lung tissue.

### Measuring N-acetyl-β-D-glucosaminidase (NAG) Activity in the Lung

NAG activity was analyzed to measure macrophage accumulation in the lung. For samples preparation, a piece of lung was diluted with 0.01 M phosphate buffer saline and homogenized (at 4°C). The resulted solutions were then frozen and thawed three times followed by 10 s sonication at room temperature to break the cells. For measurements of NAG activity, the homogenate was centrifuged (at 5,000 RPM, 30 min, 4°C). The resulted supernatants were then used to measure NAG activity using a NAGase ELISA kit following manufacturer instructions (Elabscience®, Houston, Texas, USA).

### Statistical Analysis

Data was analyzed using GraphPad Prism 7.0 (GraphPad Software, San Diego, California, USA). Values stated in the text and figures are presented as a mean ± standard error of the mean (SEM) of n observations, where n is the number of animals used. Data was tested for normality using D'Agostino-Pearon normality test and then assessed using One-way ANOVA test followed by Bonferroni's *post-hoc* test or unpaired Student *t*-test where appropriate. *P*-values of less than 0.05 were considered to be statically significant.

### Materials

Unless otherwise stated, all materials, reagents, and solutions were purchased from Sigma-Aldrich Ltd (Poole, Dorset, UK).

## Results

### Diabetic Phenotype and Characterization of Organ Dysfunction in Mice With Experimental T2DM Caused by HFD

When compared to chow fed mice, mice fed a HFD showed a significant increase in fasting blood glucose, impairment in glucose tolerance after being challenged with an oral dose of glucose, impairment in insulin tolerance after challenge with i.p. insulin, an increase in fasting plasma insulin as well as an increase in total cholesterol. Mice fed a HFD also showed significant increases in (i) total body weight secondary to an increase in fat mass, but not lean mass; (ii) an increase in the serum levels of alanine aminotransferase (ALT) indicating the development of liver injury; (iii) increase in urine albumin to creatinine ratio (ACR) as well as a (iv) a decrease in creatinine clearance (CrCl) indicating the development of diabetic nephropathy (proteinuria) and glomerular dysfunction (*P* < 0.05). Mice also showed mild cardiomyopathy as evidence by a small, but significant, reduction in ejection fraction (EF%), fractional shortening (FS%), and fractional area change (FAC%) (*P* < 0.05; Table 1).

**Table 1:** Baseline data for both chow and HFD groups before interventions (CLP or sham surgeries).

**Parameter**	**Chow**	**HFD**
Net weight gain from baseline *(grams)*	5.34 ± 0.47, *n* = 18	15.92 ± 1.19, *n* = 18^*^
Food intake$ *(grams/mouse/week)*	3.38 ± 0.02, *n* = 12 (weeks)	2.82 ± 0.03, *n* = 12 (weeks)^*^
Calorie intake$ *(Kcal/mouse/week)*	17.29 ± 0.12, *n* = 12 (weeks)	19.99 ± 0.24, *n* = 12 (weeks)^*^
AUC for OGTT *(g.min/dl)*	29.65 ± 0.55, *n* = 18	47.73 ± 2.96, *n* = 18^*^
AUC for ITT *(g.min/dl)*	6.57 ± 0.14, *n* = 5	9.63 ± 0.44, *n* = 10^*^
Fasting blood glucose *(mg/dl)*	183.9 ± 4.68, *n* = 18	294.9 ± 13.9, *n* = 18^*^
Fasting plasma insulin *(μIU/ml)*	20.16 ± 0.86, *n* = 10	71.80 ± 7.75, *n* = 9^*^
Ejection fraction *(%)*	71.92 ± 0.81, *n* = 18	64.26 ± 0.95, *n* = 18^*^
Fractional shortening *(%)*	40.73 ± 0.67, *n* = 18	34.73 ± 0.68, *n* = 18^*^
Fractional area change *(%)*	51.37 ± 0.41, *n* = 18	45.8 ± 1.14, *n* = 18^*^
Left ventricle mass *(mg)*	127.2 ± 2.92, *n* = 18	131.6 ± 4.04, *n* = 18
Urea *(mmol/L)*	9.47 ± 0.32, *n* = 18	9.45 ± 0.24, *n* = 18
Creatinine *(μmol/L)*	28.49 ± 1.71, *n* = 18	32 ± 1.25, *n* = 18
Alanine aminotransferase *(U/L)*	32.9 ± 4.05, *n* = 18	68.26 ± 9.54, *n* = 18^*^
Creatinine Clearance *(ml/min)*	154.1 ± 17.56, *n* = 14	96.55 ± 8.79, *n* = 18^*^
Urine Albumin to Creatinine Ratio *(μg/mg)*	0.19 ± 0.02, *n* = 10	0.72 ± 0.07, *n* = 10^*^
Triglyceride *(mg/dl)*	183.6 ± 9.65, *n* = 18	175.4 ± 8.53, *n* = 18
Total cholesterol *(mg/dl)*	147.3 ± 2.24, *n* = 18	183.7 ± 3.56, *n* = 18^*^

*Mice fed a HFD were compared to age-matched mice fed a chow diet. Data is presented as mean ± SEM for n number of observations. Data was analyzed by unpaired t-test*.

* P < 0.05 vs. the chow-fed group. $: mean food intake of 18 mice in each group during the study period 12 weeks (n = 12). AUC, area under the curve; OGTT, oral glucose tolerance test; ITT, insulin tolerance test

### Pre-existing T2DM Augments the Multiple Organ Dysfunction and Systemic Inflammation Associated With Sepsis in Mice

**Figure 1:**
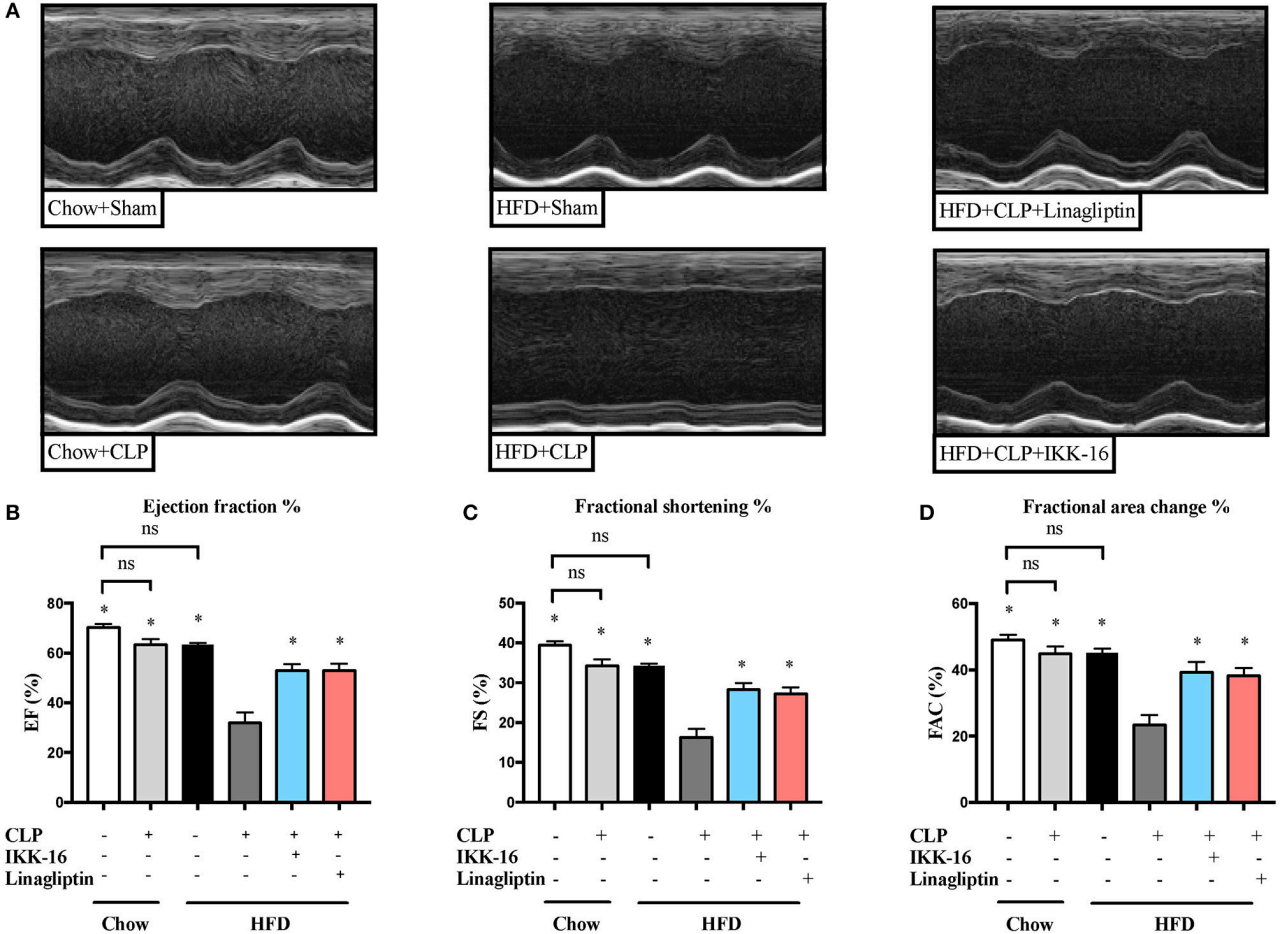
Effects of CLP challenge and linagliptin or IKK-16 post treatment on cardiac function in mice with T2DM. Mice from chow and HFD groups were randomized to undergo CLP or sham surgery. At 1 h after CLP, mice on HFD were randomized to receive linagliptin (10 mg/kg, i.v.), IKK-16 (1 mg/kg, i.v.), or vehicle (2% DMSO, 3 ml/kg). Cardiac function was assessed at 24 h after CLP or sham surgery in mice fed a chow diet or a HFD. **(A)** Representative M-mode echocardiograms; percentage % **(B)** EF, **(C)** FS, and **(D)** FAC. Data was analyzed using one-way ANOVA followed by Bonferroni's *post-hoc* test and expressed as mean ± SEM. ^*^P < 0.05 compared to HFD+CLP group. (*n* = 8 for sham surgery groups and 10 for CLP groups).

Subjecting mice on chow diet to CLP resulted in a small (but not significant) effect on systolic cardiac function compared to sham surgery (*P* > 0.05; Figure 1). However, mice fed a HFD and subjected to CLP exhibited a large and significant reduction in systolic cardiac function compared to sham surgery (*P* < 0.05; Figure 1).

**Figure 2:**
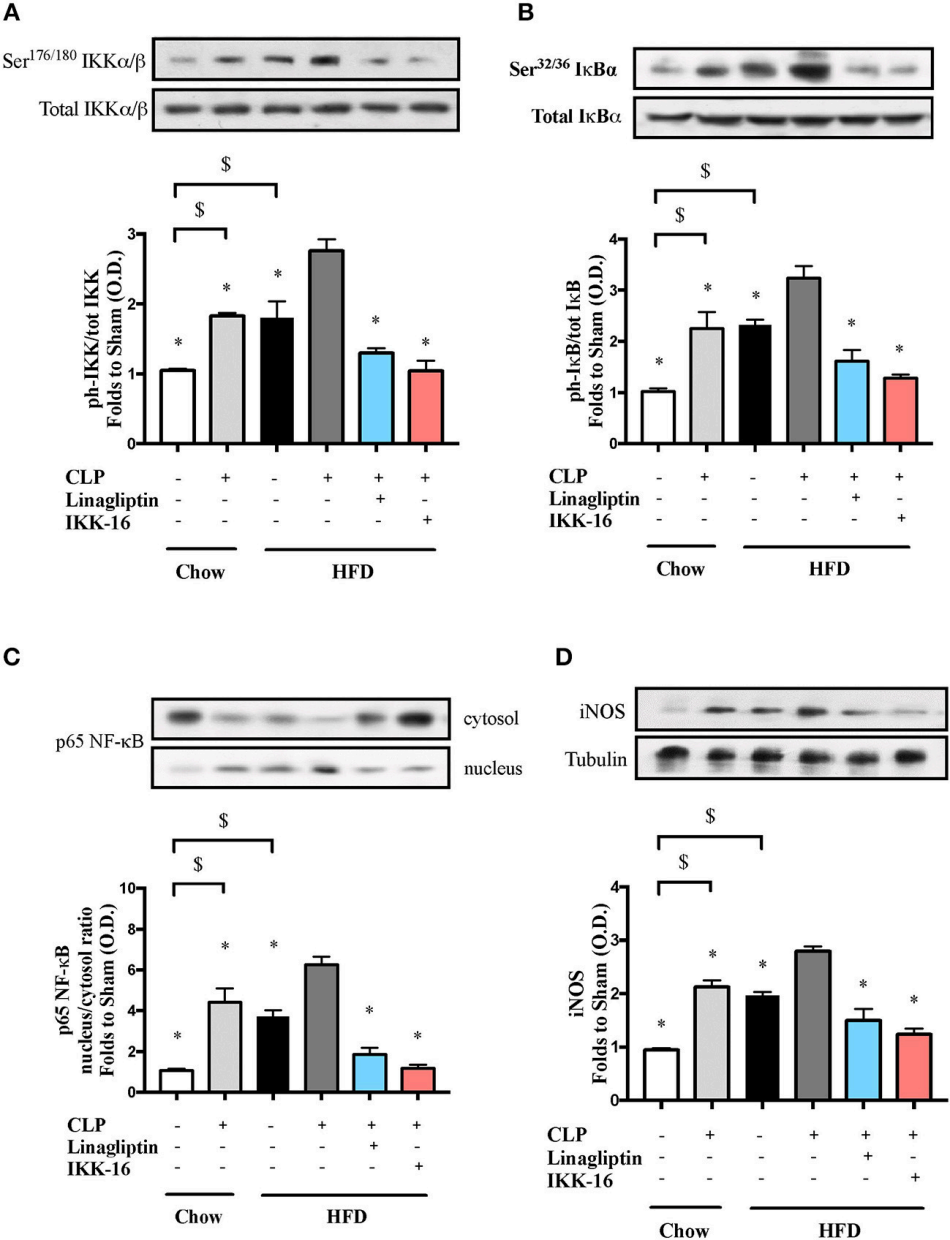
Effects of CLP and linagliptin or IKK-16 post treatment on NF-κB signaling pathway in the heart of mice with pre-existing T2DM. Mice from chow and HFD groups were randomized to undergo CLP or sham surgery. At 1 h after CLP, mice on HFD were randomized to receive linagliptin (10 mg/kg i.v.), IKK-16 (1 mg/kg i.v.), or vehicle (2% DMSO, 3 ml/kg). At 24 h heart samples were collected and signaling events were assessed. Densitometry analysis of the bands is expressed as relative optical density (O.D.) of **(A)** IKKα/β phosphorylation on Ser^178/180^ corrected to the corresponding total IKKα/β content and normalized using the related sham band; **(B)** IκBα phosphorylation on Ser^32/36^ corrected to the corresponding total IκBα content and normalized using the related sham band; **(C)** NF-κB p65 subunit levels in both, cytosolic and nuclear fractions expressed as a nucleus/cytosol ratio normalized using the related sham bands; **(D)** inducible nitric oxide synthase (iNOS) expression corrected for the corresponding tubulin band and normalized using the related sham band. Data was analyzed using one-way ANOVA followed by Bonferroni's *post-hoc* test and expressed as mean ± SEM.^*^P < 0.05 compared to HFD+CLP group, $*P* < 0.05. (*n* = 4–6 per group).

**Figure 3:**
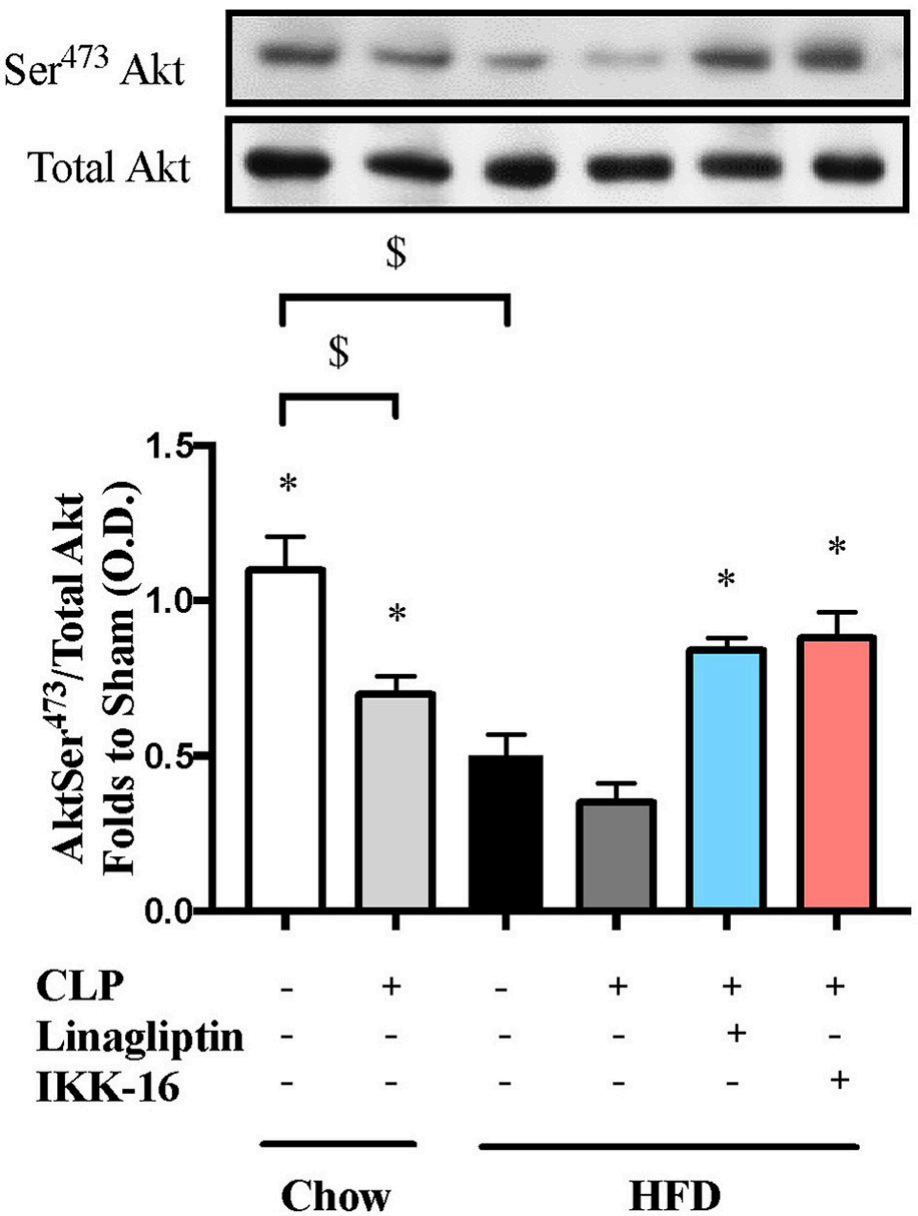
Effects of CLP and linagliptin or IKK-16 post treatment on Akt pro-survival pathway in the heart of mice with pre-existing T2DM. Mice from chow and HFD groups were randomized to undergo CLP or sham surgery. At 1 h after CLP, mice on HFD were randomized to receive linagliptin (10 mg/kg i.v.), IKK-16 (1 mg/kg i.v.), or vehicle (2% DMSO, 3 ml/kg). At 24 h, heart samples were collected and signaling events were assessed. Densitometry analysis of the bands is expressed as relative optical density (O.D.) of phosphorylated Akt on Ser^473^ corrected for the corresponding total Akt content and normalized using the related sham band. Data was analyzed using one-way ANOVA followed by Bonferroni's *post hoc* test and expressed as mean ± SEM.^*^P < 0.05 compared to HFD+CLP group, $*P* < 0.05. (*n* = 4–6 per group).

To understand the signaling mechanism associated with cardiac dysfunction, we investigated the effect of HFD on the activation of key signaling pathways of inflammation and cell survival including pathways leading to the activation of NF-κB with or without sepsis. Mice fed a HFD exhibited significant increases in the phosphorylation of IKKα/β on Ser^178/180^, the phosphorylation of IκBα on Ser^32/36^, the translocation of p65 NF-κB to the nucleus, the expression of iNOS, and a significant decrease in the phosphorylation of Akt on Ser^473^ (*P* < 0.05; Figures 2, 3). Exposing mice on chow diet to CLP resulted in a similar degree of activation of NF-κB and, indeed, iNOS expression (in the heart) as observed in mice with HFD alone. CLP-sepsis in mice on HFD resulted in a further increase in the phosphorylation of IKKα/β and IκBα, the translocation of p65, and iNOS expression (*P* < 0.05; Figure 2) with no significant effect on Akt phosphorylation (*P* > 0.05; Figure 3).

**Figure 4:**
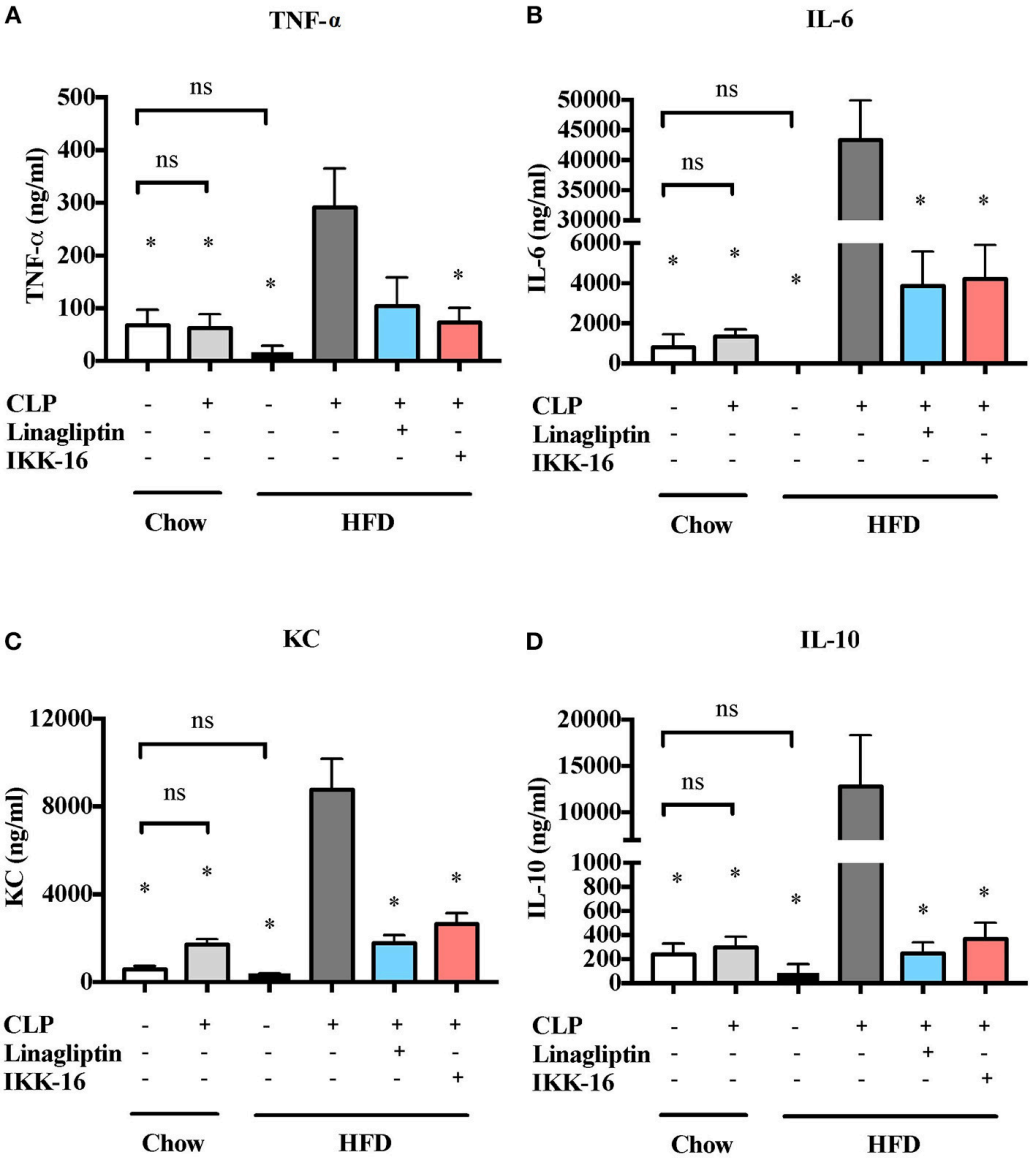
Effects of CLP challenge and linagliptin or IKK-16 post treatment on systemic inflammation in mice with T2DM. Mice from chow and HFD groups were randomized to undergo CLP or sham surgery. At 1 h after CLP, mice on HFD were randomized to receive linagliptin (10 mg/kg, i.v.), IKK-16 (1 mg/kg, i.v.), or vehicle (2% DMSO, 3 ml/kg). At 24 h after CLP, blood samples were collected and inflammatory cytokines concentrations were measured in the serum. **(A)** TNF-α, **(B)** IL-6, **(C)** KC, and **(D)** IL-10. Data was analyzed using one-way ANOVA followed by Bonferroni's *post-hoc* test and expressed as mean ± SEM. ^*^P < 0.05 compared to HFD+CLP group. (*n* = 4 per sham surgery group and *n* = 6–8 per CLP group).

We also studied the effect of HFD on the systematic synthesis of key, NF-κB-dependent cytokines including TNF-α, IL-6, KC, and IL-10. When compared to mice on chow diet, mice on HFD for 12 weeks showed no significant changes in cytokines levels (*P* > 0.05; Figure 2). When compared to mice on regular chow diet subjected to sham surgery, mice on chow diet subjected to CLP (in the presence of antibiotics) showed no significant changes in cytokines levels in the serum (*P* > 0.05; Figure 4). CLP-sepsis in mice on HFD resulted in large increases in the serum levels of TNF-α, IL-6, KC, and IL-10 when compared to mice on HFD subjected to sham surgery and mice on chow diet subjected to CLP (*P* < 0.05; Figure 4).

**Figure 5:**
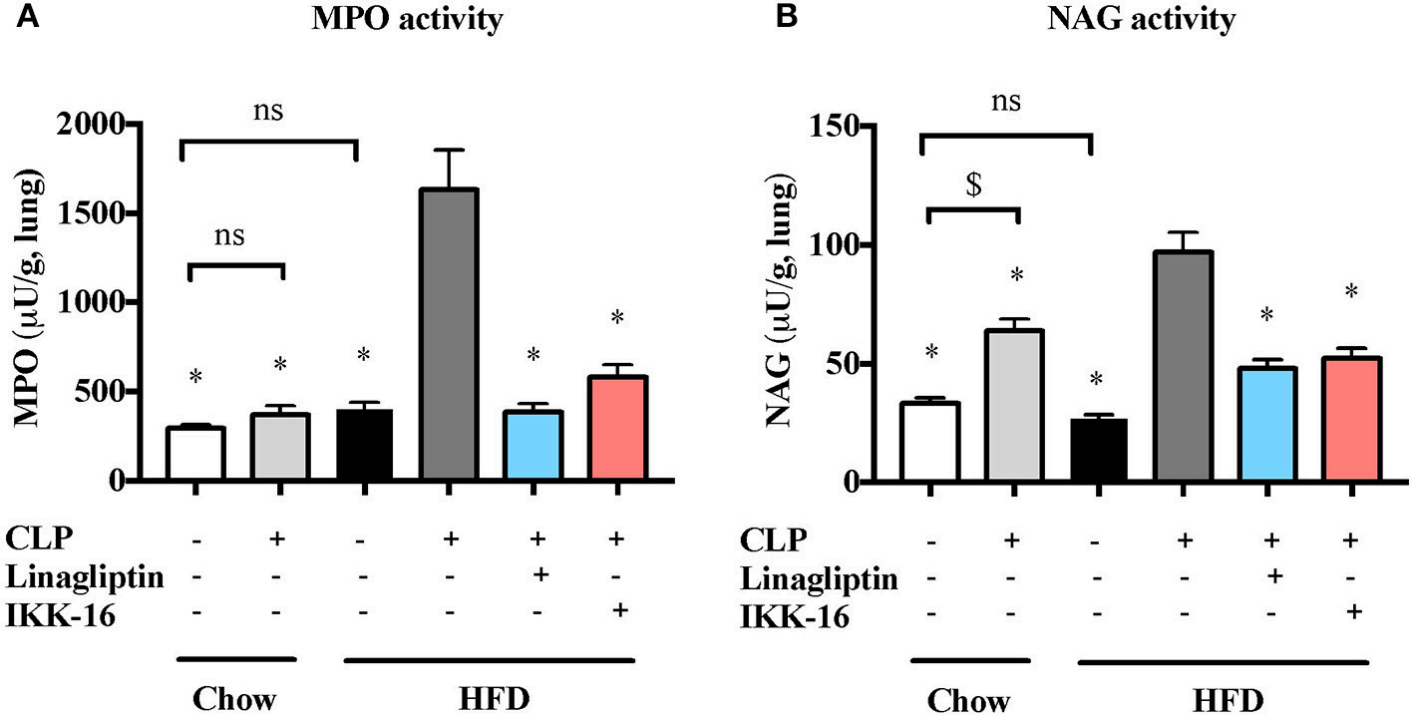
Effects of CLP and linagliptin or IKK-16 post treatment on neutrophil/macrophage infiltration in the lung in mice with pre-existing T2DM. Mice from chow and HFD groups were randomized to undergo CLP or sham surgery. At 1 h after CLP, mice on HFD were randomized to receive linagliptin (10 mg/kg, i.v.), IKK-16 (1 mg/kg, i.v.), or vehicle (2% DMSO, 3 ml/kg). At 24 h after CLP, lung samples were collected and neutrophil and macrophages infiltration were measured as the **(A)** MPO and **(B)** NAG activities. Data was analyzed using one-way ANOVA followed by Bonferroni's *post-hoc* test and expressed as mean ± SEM for n number of observations. ^*^P < 0.05 compared HFD+CLP group. $*P* < 0.05 (*n* = 6 per group).

**Figure 6:**
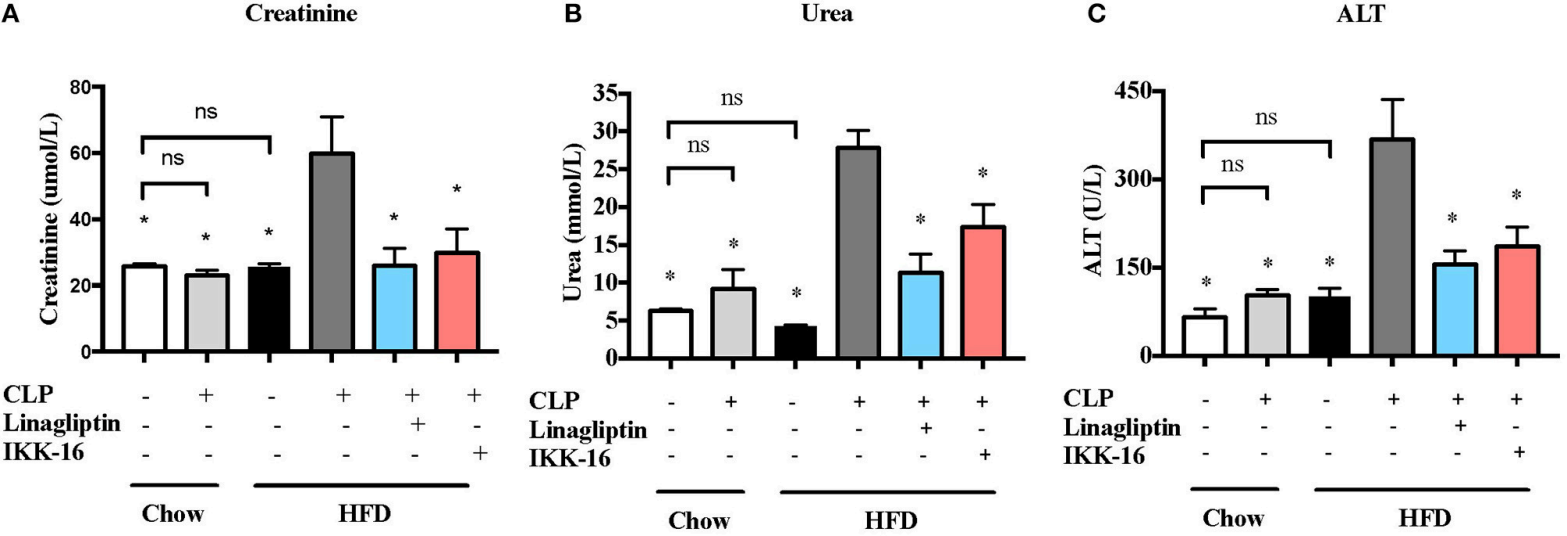
Effects of CLP and linagliptin or IKK-16 post treatment on renal dysfenction and hepatocelleular injury in mice with pre-existing T2DM. Mice from chow and HFD groups were randomized to undergo CLP or sham surgery. At 1 h after CLP, mice on HFD were randomized to receive linagliptin (10 mg/kg, i.v.), IKK-16 (1 mg/kg, i.v.), or vehicle (2% DMSO, 3 ml/kg). At 24 h after CLP, blood samples were collected and serum **(A)** urea, **(B)** creatinine, and **(C)** ALT levels were measured. Data was analyzed using one-way ANOVA followed by Bonferroni's *post-hoc* test and expressed as mean ± SEM. ^*^P < 0.05 compared to HFD+CLP group. (*n* = 8 per sham surgery group, *n* = 10 per CLP groups).

Markers for lung inflammation were also measured to study the effect of pre-existing diabetes on lung injury associated with sepsis. When compared to micev on chow diet, mice on HFD showed no significantv changes in MPO or NAG activities in the lungs after sham surgery (*P* > 0.05; Figure 5). Whenv compared to mice on regular chow diet subjected to sham surgery, mice on chow diet subjected to CLP showed a significant increase in NAG activity in the lung (*P* < 0.05; Figure 5) with no change in MPO activity (*P* > 0.05; Figure 6). Subjecting mice on HFD to CLP surgery resulted in big increases in both MPO and NAG activities when compared to mice on HFD subjected to sham surgery and mice on chow diet subjected to the same CLP (*P* < 0.05; Figure 5).

Our CLP-sepsis model with moderate severity had (in the presence of antibiotics and fluid resuscitation) no effect on serum creatinine, urea, or ALT compared to sham surgery (*P* > 0.05; Figure 6). However, challenge of mice on HFD with CLP resulted (despite the presence of antibiotics and fluid resuscitation) in significant increases in serum creatinine, urea, and ALT levels (*P* < 0.05; Figure 6). These results indicate that pre-existing T2DM increases the severity of both renal dysfunction and hepatocellular injury.

### Effect of Linagliptin Post Treatment on the Multiple Organ Dysfunction Associated With Sepsis in Mice With Pre-existing T2DM

When compared to mice on HFD subjected to sham surgery, mice on HFD subjected to CLP and treated with vehicle developed significant systolic cardiac dysfunction. Treatment of mice on HFD with linagliptin, at 1 h after CLP, attenuated the systolic cardiac dysfunction caused by CLP (*P* < 0.05; Figure 1).

Having found that linagliptin attenuates the cardiac dysfunction associated with CLP-sepsis in diabetic mice, we then investigated the potential mechanism(s) of this beneficial effect. Treatment of mice on HFD with linagliptin, at 1 h after CLP, also resulted in significant reduction in IKKα/β and IκBα phosphorylation, p65 translocation, and iNOS expression when compared to mice on HFD subjected to CLP and treated with vehicle (*P* < 0.05; Figure 2). Moreover, linagliptin treatment of HFD/CLP mice restored the degree of Akt phosphorylation to almost that seen in sham mice (*P* < 0.05; Figure 3).

Systemic inflammation was also attenuated after linagliptin treatment. When compared to mice on HFD challenged CLP and treated with vehicle, delayed treatment with linagliptin at 1 h after CLP resulted in significant reduction in IL-6, KC, and IL-10 synthesis (*P* < 0.05; Figure 4) and in a reduction in TNF-α that did not, however, reached statistical significance when compared to mice treated with vehicle (*P* > 0.05, Figure 4).

Accordingly, a significant reduction in markers of lung inflammation, specifically the MPO and NAG activities, was observed in the lungs of mice exposed to linagliptin post-treatment when compared to mice on HFD subjected to CLP and treated with vehicle only (*P* < 0.05, Figure 5).

When compared to mice on HFD subjected to CLP and treated with vehicle, mice on HFD and treated with linagliptin, at 1 h after CLP, showed significant reduction in serum creatinine, urea, and ALT levels indicating that linagliptin attenuated the renal dysfunction and liver injury caused by CLP (*P* < 0.05; Figure 6).

### Effect of IKK-16 Post Treatment on the Multiple Organ Dysfunction Associated With Sepsis in Mice With Pre-existing T2DM

In order to investigate whether inhibition of NF-κB was the main reason of the attenuated organ injury caused by sepsis in diabetic mice after linagliptin treatment, we have investigated the effects of the specific IKK-inhibitor IKK-16 in these animals. When compared to mice on HFD subjected to sham surgery, mice on HFD subjected to CLP and treated with vehicle developed significant systolic cardiac dysfunction. Delayed treatment of HFD mice with IKK-16 at 1 h after CLP attenuated the systolic cardiac dysfunction (*P* < 0.05; Figure 1) caused by sepsis.

The restoration of cardiac function afforded by IKK-16 in diabetic CLP-mice was accompanied by significant reduction in IKKα/β and IκBα phosphorylation, p65 translocation, and iNOS expression in mice treated with IKK-16 when compared to mice on HFD challenged with CLP and treated with vehicle (*P* < 0.05; Figure 2). Moreover, IKK-16 treatment restored the Akt phosphorylation (*P* < 0.05; Figure 3) caused by HFD with or without sepsis.

When compared to mice on HFD subjected to CLP and treated with vehicle, delayed treatment with IKK-16 at 1 h after CLP resulted in significant reduction of both the systemic levels TNF-α, IL-6, KC, and IL-10 (*P* < 0.05; Figure 4) and local (lung) MPO and NAG activities (*P* < 0.05, Figure 5). They also showed significant reduction of serum creatinine, urea, and ALT levels indicating that IKK-16 reduced both the renal dysfunction and the liver injury caused by sepsis in mice fed with a HFD (*P* < 0.05; Figure 6).

Interestingly, no statistically significant differences between the two post-treatment groups were recorded for any of the measured markers.

## Discussion

Although the mortality rate among septic patients has declined due to improved supportive care for patients in the ICU (39), the incidence of sepsis has increased as a result of the aging of the population (40) which is associated with the presence of significant comorbidities such as T2DM (5). Patients with T2DM are more likely to develop infections and subsequently sepsis (10, 11). The cardiovascular system is one of the most important systems affected by sepsis and the development of cardiovascular dysfunction in sepsis has been linked to several pathophysiological driver including inflammatory cytokines and NO (32, 36). Many studies of the pathophysiology of sepsis have shown beneficial effects in pre-clinical models of sepsis. However, clinical studies that tested the efficacy of drugs targeted at identical aspects of the pathophysiology (often by using identical interventions) have failed to improve survival in septic patients (as a result of the limitations in both animal models and experimental designs) (41).

In this study, a clinically relevant model of T2DM caused by prolonged administration of HFD (for 12 weeks) was established in C57BL/6 male mice. As the consumption of a western diet is a key driver underlying the development of T2DM, our model of feeding a HFD for longer periods recapitulates the main cause of T2DM in humans and is considered to be one of the most clinically, relevant model of T2DM to date. Indeed, feeding of mice with HFD resulted, within 12 weeks, in the development of a diabetic phenotype (significant weight gain, impaired glucose tolerance, increased fasting blood glucose and increased fasting plasma insulin) and diabetic cardiomyopathy (reduction in %EF) as a result of NF-κB activation in the heart (see below).

A “two-hit” model of pre-existing T2DM (secondary to HFD administration) followed by a mild CLP surgery (which did not cause significant organ dysfunction in young and healthy mice, but which we have reported to cause substantial organ dysfunctions in old mice or mice with CKD) was used to study (a) the effect of pre-existing T2DM on the cardiac dysfunction associated with sepsis and (b) to test novel therapeutic interventions aimed at reducing cardiac dysfunction in T2DM/sepsis mice. We show here, for the first time, that pre-existing T2DM augments the cardiac dysfunction associated with sepsis. T2DM alone resulted in a small degree of NF-κB activation and iNOS expression in the heart. However, sepsis (second hit) in diabetic mice resulted in a dramatic increase in the serum concentrations of proinflammatory cytokines and a further increase in both NF-κB activation and iNOS expression in the heart. Diabetes also resulted in reduction in the activation (phosphorylation) of the Akt pro-survival pathway, while sepsis resulted in further reduction of Akt phosphorylation in the heart. These two findings suggest that the cardiac dysfunction associated with T2DM/sepsis is most likely a result of the activation of the NF-κB pro-inflammatory signaling pathway (with subsequent increase in iNOS expression and serum inflammatory cytokines levels) and the concomitant inhibition of Akt pro-survival pathways.

The DPP-4 inhibitors gliptins (e.g., linagliptin and sitagliptin) have been used as anti-diabetic drugs that exert their catalytic effect by increasing the incretin levels. However, recent evidence indicates that DPP-4 inhibitors, as well as glucagon like peptide-1 (GLP-1) receptors agonists (e.g., liraglutide), also have anti-inflammatory effects. The (off-target) non-catalytic effects of DPP-4 inhibitors have recently been discussed in the literature as a potential new therapeutic strategy for the treatment of diseases associated with local or systemic inflammation. Indeed, some preclinical studies suggest that inhibition of DPP-4 by different gliptins results in less cardiac dysfunction in a murine model of HFD-induced fibrosis and inflammation (42) and in a rat model of heart failure (43) by inhibiting NF-κB and by reducing the formation of pro-inflammatory cytokines. The effect of some gliptins and GLP-1 receptor agonists on survival and inflammation was also studied in animal models of endotoxaemia. Rodents subjected to endotoxaemia and treated with linagliptin or liraglutide (or their respective vehicles) showed an increase in survival rate (44, 45), and decreased formation of reactive oxygen species (ROS) (46). Treatment of cardiomyocytes with sitagliptin decreased the inflammatory response triggered by LPS (47). In contrast, pre-treatment with sitagliptin had no effect on survival in endotoxaemic animals (44, 45). Furthermore, treatment of endotoxaemic mice with vildagliptin ameliorated the degree of pulmonary fibrosis (48). However, the effect of DPP-4 inhibitors on the cardiac (organ) dysfunction associated with sepsis (in the absence or presence of diabetes) has not yet been investigated.

Based on previous work in animals with sepsis (without T2DM), we have hypothesized that the activation of NF-κB (pro-inflammatory) and the inhibition of Akt (pro-survival) pathways are the reasons for the cardiac dysfunction in T2DM/sepsis and, hence, studied the effect of linagliptin (repurposing of linagliptin) on NF-κB inhibition and Akt activation and their impact on cardiac performance. Indeed, the results of this study highlighted, for the first time, that inhibition of DPP-4 using linagliptin (at 1 h after CLP) attenuates the cardiac dysfunction associated with T2DM/CLP-sepsis and this was associated with, or occurred as a result of, an inhibition of NF-κB activation and preservation of Akt pathway activation in the heart. Treatment with linagliptin also resulted in attenuation of the multiple organ dysfunction associated with T2DM/CLP-sepsis.

To confirm the potential key role of the activation of NF-κB in the pathophysiology of septic cardiomyopathy in animals with T2DM, we investigated the effect of NF-κB pathway inhibition using a selective IKK inhibitor (IKK-16) on cardiac (organ) dysfunction associated with sepsis. Treatment with IKK-16 ameliorated the cardiac dysfunction in mice with T2DM/sepsis. This enhanced cardiac function was a result of the decreased NF-κB activation (and iNOS expression) and inflammatory cytokines synthesis and the restored Akt phosphorylation. This restoration of Akt phosphorylation can be a result of heat shock protein 90 (Hsp 90) binding to the α and β subunits of IKK as well as to endothelial nitric oxide synthase (eNOS) (49, 50). The interaction of Hsp90 and eNOS creates a complex with Akt which allows eNOS and Akt to function on the same domain of Hsp90 (51). This interaction is increased when IKK is inhibited, resulting in increased Akt-eNOS pathway activation (52). Many other studies showed that pharmacological interventions that inhibit NF-κB reduced the multiple organ dysfunction associated with sepsis (32, 36, 53). Indeed, in this study a single dose of IKK-16 at 1 h after CLP resulted in attenuation of the multiple organ dysfunction associated with T2DM/sepsis.

Although we cannot exclude that other effects associated with DPP-4 inhibition (that do not involve NF-κB inhibition) may have contributed to the observed beneficial effects of linagliptin, our data demonstrating that the magnitude of the effect of the inhibition of NF-κB with IKK-16 is similar to the one observed with linagliptin supports the view that inhibition of the activation of NF-κB is at the heart of the observed beneficial effects of both linagliptin and IKK-16. Our data also indicate (and indeed support the view of other publications) that linagliptin may be “repurposed” for the use in sepsis and/or other conditions that are associated with local or systemic inflammation driven by the excessive activation of NF-κB.

## Conclusions and Limitations

Our data shows that a pre-existing, diabetic phenotype worsens the cardiac (organ) dysfunction associated with CLP-sepsis in mice. It also shows that activation of the NF-κB pathway is a key driver of cardiac dysfunction in mice with T2DM/sepsis. Most notably, it shows, for the first time, that inhibition of NF-κB using linagliptin or IKK-16 attenuates this cardiac (organ) dysfunction even in mice with pre-existing T2DM. Therefore, targeting NF-κB activation may be a potential strategy to treat the excessive inflammation and cardiac (organ) dysfunction in patients with T2DM and sepsis. However, our study was conducted in relatively young mice (22-week old) and treatment of septic mice was introduced relatively early in the disease course (at 1 h of the induction of sepsis) in mice treated with antibiotics and fluids to mimic “standard care” in humans with sepsis. Clearly, more studies are needed to elucidate how late after the onset of sepsis linagliptin or IKK-16 can be administered to attenuate the cardiac (organ) dysfunction, especially in older mice (ideally 12 to 18-month old). The latter studies are often limited by either the availability of mice of a relevant age or cost of these animals (and often both). This is of particular importance as most cases of sepsis occur in elderly and they are usually diagnosed later in the disease when patients either have already developed multiple organ dysfunctions or, at least, significant cardiovascular abnormalities. Further studies using other DPP-4 inhibitors and/or GLP-1 receptor agonists are needed to investigate whether any of the observed beneficial effects of linagliptin are secondary to the increased incretin levels or are, indeed, a direct effect of DPP-4 inhibition and to determine whether the inhibition of NF-κB reported with linagliptin in our study is, indeed, a unique class-specific effect or an off-target effect of linagliptin.

## Author Contributions

SA, JC, MC, MY, and CT conceived and designed the experiment. SA, JC, CM, LM, FC, and DC performed the experiments. SA, MC, CT analyzed the data. SA, MC, and CT contributed to the writing of the manuscript.

### Conflict of Interest Statement

The authors declare that the research was conducted in the absence of any commercial or financial relationships that could be construed as a potential conflict of interest.
